# Machine Learning to Detect Self-Reporting of Symptoms, Testing Access, and Recovery Associated With COVID-19 on Twitter: Retrospective Big Data Infoveillance Study

**DOI:** 10.2196/19509

**Published:** 2020-06-08

**Authors:** Tim Mackey, Vidya Purushothaman, Jiawei Li, Neal Shah, Matthew Nali, Cortni Bardier, Bryan Liang, Mingxiang Cai, Raphael Cuomo

**Affiliations:** 1 Department of Anesthesiology and Division of Global Public Health and Infectious Diseases School of Medicine University of California San Diego La Jolla, CA United States; 2 Global Health Policy Institute San Diego, CA United States; 3 S-3 Research LLC San Diego, CA United States; 4 Department of Healthcare Research and Policy University of California San Diego San Diego, CA United States; 5 Department of Family Medicine and Public Health School of Medicine University of California San Diego La Jolla, CA United States; 6 Masters Program in Global Health Department of Anthropology University of California San Diego La Jolla, CA United States; 7 Masters Program in Computer Science Jacobs School of Engineering University of California San Diego La Jolla, CA United States

**Keywords:** infoveillance, COVID-19, Twitter, machine learning, surveillance

## Abstract

**Background:**

The coronavirus disease (COVID-19) pandemic is a global health emergency with over 6 million cases worldwide as of the beginning of June 2020. The pandemic is historic in scope and precedent given its emergence in an increasingly digital era. Importantly, there have been concerns about the accuracy of COVID-19 case counts due to issues such as lack of access to testing and difficulty in measuring recoveries.

**Objective:**

The aims of this study were to detect and characterize user-generated conversations that could be associated with COVID-19-related symptoms, experiences with access to testing, and mentions of disease recovery using an unsupervised machine learning approach.

**Methods:**

Tweets were collected from the Twitter public streaming application programming interface from March 3-20, 2020, filtered for general COVID-19-related keywords and then further filtered for terms that could be related to COVID-19 symptoms as self-reported by users. Tweets were analyzed using an unsupervised machine learning approach called the biterm topic model (BTM), where groups of tweets containing the same word-related themes were separated into topic clusters that included conversations about symptoms, testing, and recovery. Tweets in these clusters were then extracted and manually annotated for content analysis and assessed for their statistical and geographic characteristics.

**Results:**

A total of 4,492,954 tweets were collected that contained terms that could be related to COVID-19 symptoms. After using BTM to identify relevant topic clusters and removing duplicate tweets, we identified a total of 3465 (<1%) tweets that included user-generated conversations about experiences that users associated with possible COVID-19 symptoms and other disease experiences. These tweets were grouped into five main categories including first- and secondhand reports of symptoms, symptom reporting concurrent with lack of testing, discussion of recovery, confirmation of negative COVID-19 diagnosis after receiving testing, and users recalling symptoms and questioning whether they might have been previously infected with COVID-19. The co-occurrence of tweets for these themes was statistically significant for users reporting symptoms with a lack of testing and with a discussion of recovery. A total of 63% (n=1112) of the geotagged tweets were located in the United States.

**Conclusions:**

This study used unsupervised machine learning for the purposes of characterizing self-reporting of symptoms, experiences with testing, and mentions of recovery related to COVID-19. Many users reported symptoms they thought were related to COVID-19, but they were not able to get tested to confirm their concerns. In the absence of testing availability and confirmation, accurate case estimations for this period of the outbreak may never be known. Future studies should continue to explore the utility of infoveillance approaches to estimate COVID-19 disease severity.

## Introduction

As of the beginning of June 2020, the novel coronavirus disease (COVID-19) pandemic has now reached over 6 million confirmed cases worldwide (over 1.7 million in the United States alone) and approximately 370,000 deaths worldwide according to the Johns Hopkins University Coronavirus Resource Center. COVID-19 case counts are alarming in both their volume and widening geographic scope. There are also concerns about the accuracy of reported COVID-19 case counts, particularly at earlier stages of the pandemic, and whether underreporting may have obscured the true extent of the outbreak, its underlining epidemiological characteristics, and its overall health and societal impact [1-3].

Specifically, concerns regarding COVID-19 underreporting are influenced by factors such as lack of access to testing kits; a lag in reporting and registering cases due to overburdened health systems; failure to report or test before or after a COVID-19-suspected death; variation in testing administration or decision making (eg, foregoing testing when it would not change the course of treatment for a patient); and uncomplicated, mild, or asymptomatic cases simply never being tested or seeking care [4,5]. Concerns about underreporting have been pervasive, with media reports highlighting challenges in countries with outbreaks of different scale and at varying time periods, including the United States, China, Iran, and Russia, to name a few [6-9].

Accurate estimations of the number of people who have recovered from COVID-19 are also difficult to ascertain. The John Hopkins University Coronavirus Resource Center COVID-19 data dashboard is one source that aggregates the number of reported COVID-19 recovered cases, which now stands at over 2.6 million worldwide. However, case reporting on recoveries can be difficult to measure and define, leading to potential overestimation of the mortality rate and underestimation of community spread that can complicate efforts toward estimating population immunity [5]. Reflecting these challenges, COVID-19 recovered cases are often limited to data aggregated at the country or national level, are derived only from confirmed cases, and may differ based on the definition of “recovery” or method of confirmation [5,10].

In response, this study sought to better understand the characteristics of publicly available self-reported user-generated conversations associated with terms that could be related to COVID-19 symptoms, recoveries, and testing experiences. This was accomplished using a retrospective observational infoveillance study during earlier stages of the global pandemic. Infoveillance studies, which use data from the internet, social media, and other information in an electronic medium for disease surveillance purposes, have been used in prior outbreaks (eg, H1N1, Ebola) [11-15]. There is also an emerging base of literature using social media and website search results to explore the COVID-19 pandemic [16-21].

## Methods

This retrospective infoveillance study was conducted in two phases: (1) data collection using the public streaming Twitter application programming interface (API) for COVID-19-related keywords; and (2) data cleaning, processing, and analysis of tweets using an unsupervised machine learning approach by means of natural language processing, followed by subsequent statistical and geospatial analysis of twitter message characteristics.

We first collected tweets by filtering for general COVID-19-related keywords including: “covid19,” “corona,” “coronavirus,” “coronavid19.” Following the collection of a corpus of general COVID-19 tweets, we further filtered this corpus for terms that could be associated with COVID-19 symptoms, testing, and recovery conversations. These additional terms included: “diagnosed,” “pneumonia,” “fever,” “test,” “testing kit,” “sharing,” “symptoms,” “isolating,” “cough,” “ER” (emergency room), and “emergency room.” The COVID-19-related keywords were chosen based on relevance to general COVID-19 social media conversations as used in prior studies [16,18,22]. Filtered terms were chosen based on manual searches conducted by the study team prior to the commencement of the study, where user-generated tweets associated with COVID-19 symptoms were detected and the terms used were assessed.

Data was collected from the Twitter public API from March 3-20, 2020. For data processing, we first removed hashtags, stop words, and the top 100 news Twitter handles or accounts. We removed the top news accounts as the focus of this study was on user-generated content, both first- and secondhand accounts, of COVID-19 experiences, not COVID-19 news and media sources of information.

For data analysis, we used the biterm topic model (BTM), an unsupervised machine learning approach to extract themes from groups of texts as used in prior studies to detect substance abuse disorder and other public health issues [23-25]. Groups of messages or text containing the same word-related themes are categorized into clusters; the main themes of those clusters are considered as the topic of the text aggregation, which is then split into a bag of words where a discrete probability distribution for each theme is generated [26]. Using BTM, we identified topic clusters with word groupings, frequencies, and characteristics that appeared to be related to symptoms, recovery, and testing experiences with COVID-19 (“signal”) and then extracted tweets from these topic clusters for manual annotation.

The number of topic clusters we chose to extract (k) can affect the results associated with these topics. Too many clusters could lead to diffusion of signal, while too few clusters may conceal possible signals in the topics. To address this, we used a coherence score to measure the quality of the number of topics we chose by measuring how correlated the texts are in the same clusters. A higher coherence score means the text in the cluster are more correlated to each other. We chose five different k values for the number of clusters (k=5,10,15,20,25), then we calculated the coherence score and identified the k value with the highest score as a parameter for BTM.

Here is how we calculate the u-mass coherence score C(t;*v^t^*). We let D(v) be the document frequency of the word type v (ie, the number of documents containing at least one token of type v) and D(v, v0) be the codocument frequency of word types v and v0 (ie, the number of documents containing one or more tokens of type v and at least one token of type v0). We define topic coherence as:


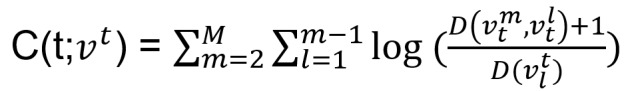


V(t) = 
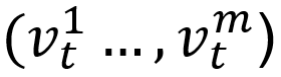
 is a list of the M most probable words in topic t. A smoothing count of 1 is included to avoid taking the logarithm of zero.

Manual annotation of tweets was conducted by authors VP, NS, MN, and CB. Coding was focused on content analysis using an inductive coding scheme, including a binary classification of whether the tweet discussed symptoms that could be related to COVID-19 (including firsthand or secondhand accounts), experiences with seeking COVID-19 testing access, or disease recovery, and the co-occurrence of these themes (see Multimedia Appendix 1 for description of coding schema). VP, NS, MN, and CB coded posts independently and achieved high intercoder reliability (kappa=0.98). For inconsistent results, authors reviewed and conferred on correct classification with author TM.

Data collection and analysis was conducted using the Python (Python Software Foundation) programming language and associated package Tweepy. Statistical and geospatial analysis was carried out using RStudio 3.6.1 (RStudio, Inc) and ArcGIS (Esri). For statistical analysis and geospatial visualization, COVID-19 cases from March 20, 2020, were obtained from the JHU GitHub CSSEGISandData file.

Ethics approval and consent to participate was not required for this study. All information collected from this study was from the public domain, and the study did not involve any interaction with users. Users’ indefinable information was removed from the study results.

## Results

A total of 72,922,211 tweets were collected from March 3-20, 2020, from the Twitter public API filtered for general COVID-19-related keywords. From this entire corpus, we filtered for the previously mentioned additional terms associated with COVID-19 symptoms, testing, and recovery conversations, resulting in a filtered data set of 4,492,954 tweets (ie, this data set included tweets with both COVID-19 general terms and at least one additional term). BTM was then used to analyze the filtered data set to identify relevant topic clusters. After identifying topic clusters that had characteristics related to signal, we extracted 35,786 tweets contained in these BTM topic clusters for the purposes of manual annotation (ie, this data set represents all tweets that were contained in relevant BTM topic clusters selected for manual labelling). After removing duplicates and manually annotating tweets, 3465 (0.00077% of the filtered data set) posts from 2812 unique users were confirmed and identified as signal conversations related to symptoms, testing experiences, or recovery that users associated with COVID-19 (ie, this data set represents true positives that were identified by manual annotation).

Signal tweets were grouped into five main thematic categories: (1) firsthand and secondhand (eg, family, friends) reporting of suspected symptoms that users associated with COVID-19 (eg, fever, cough, shortness of breath, chills); (2) symptom reporting with concurrent discussion of lack of access to COVID-19 testing, mostly due to rigorous criteria to qualify for testing (eg, symptom severity, fever, travel history, insurance) and with no confirmatory diagnosis; (3) user discussion of recovery from suspected COVID-19 symptoms; (4) user confirmation of a negative COVID-19 diagnosis after receiving testing; and (5) users recalling symptoms in the past 5 months that they suspected as possibly associated with a COVID-19 infection (see deidentified examples in Table 1).

Metadata associated with users from these signal tweets indicated that the majority of these conversations were most likely organic (ie, originating and consisting of user-generated content). Though we did not explicitly filter our tweets for bot or spam traffic, the average ratio of users’ followers to following was 1607:78, and only 111 users had accounts created recently in 2020. We also observed during our manual coding that these accounts generally included longer interactions with other users, original content, and profile information that had individually identifiable information or biographies. Generally, these user metadata characteristics are reflective of organic content versus automated and social bot-based content.

**Table 1:** Numbers and examples of posts related to COVID-19 symptoms, access to testing, and recovery (modified for deidentification; n=3465).

Theme^a^		Posts, n (%)^b^	Example conversation^c^
**Conversations about symptoms**			
	Self-reporting of symptoms (firsthand) Secondhand reporting of symptoms	3465 (100)	“*1/I went to ER*^d^ *day before Asked by Dr why I was there I said “I have Coronavirus symptoms.” (I really do.) He laughed; asked what symptoms were. I gave all the Coronavirus symptoms. He said “I believe you have an upper respiratory virus. Let’s give you a steroid shot.”* “*Contacted the er and [FACILITY NAME] in [CITY] because my daughter has a runny nose fever and a sore throat. I was told they’re testing for everything else before testing for coronavirus. Is that backwards or am I trippin? #CoronaVirusSeattle”*
**Conversations about symptoms concurrent with other themes**			
	Symptom reporting and lack of access to testing	512 (14.8)	“*Hey [NAME] why can’t we get tested for COVID-19*^e^ *in [LOCATION]? My wife has all the symptoms but ER said no testing unless you’re admitted.”*
	Conversations about symptom and recovery User confirmation not COVID-19 case after testing User recalling past COVID-19 suspected symptoms	780 (22.5)	“*My spouse, 4 yr old and I are almost better now. We were sick about ten days. Don’t know if it is Corona because we could not get a test. Fever lasted 3 to 4 days. No cough for us. Consistent headache, chills, sore throat. Reduced appetite for a few days Hydrate! Nap! ”* “*I went to the doctor and they contacted the CDC*^f^ *thinking it was Coronavirus and tried to quarantine me when it was just the flu (I was tested at the ER and it’s NOT) thank you to the [NAME] nurse and clinic for being very misinformed & freaking me out* 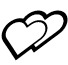 *”* “*Just before Christmas I was diagnosed with pneumonia. In acute pain breathing I had cough that wouldn’t go away for weeks &; was so fatigued I slept for hours every day. I had no appetite or the strength. It lasted for approx 2 weeks. Was it #coronavirus”*

^a^Discrete or concurrent signal.

^b^Number of posts and the percentage of total signal posts that contained the theme.

^c^Twitter posts or comments with signal.

^d^ER: emergency room.

^e^COVID-19: coronavirus disease.

^f^CDC: Centers for Disease Control and Prevention.

**Figure 1:**
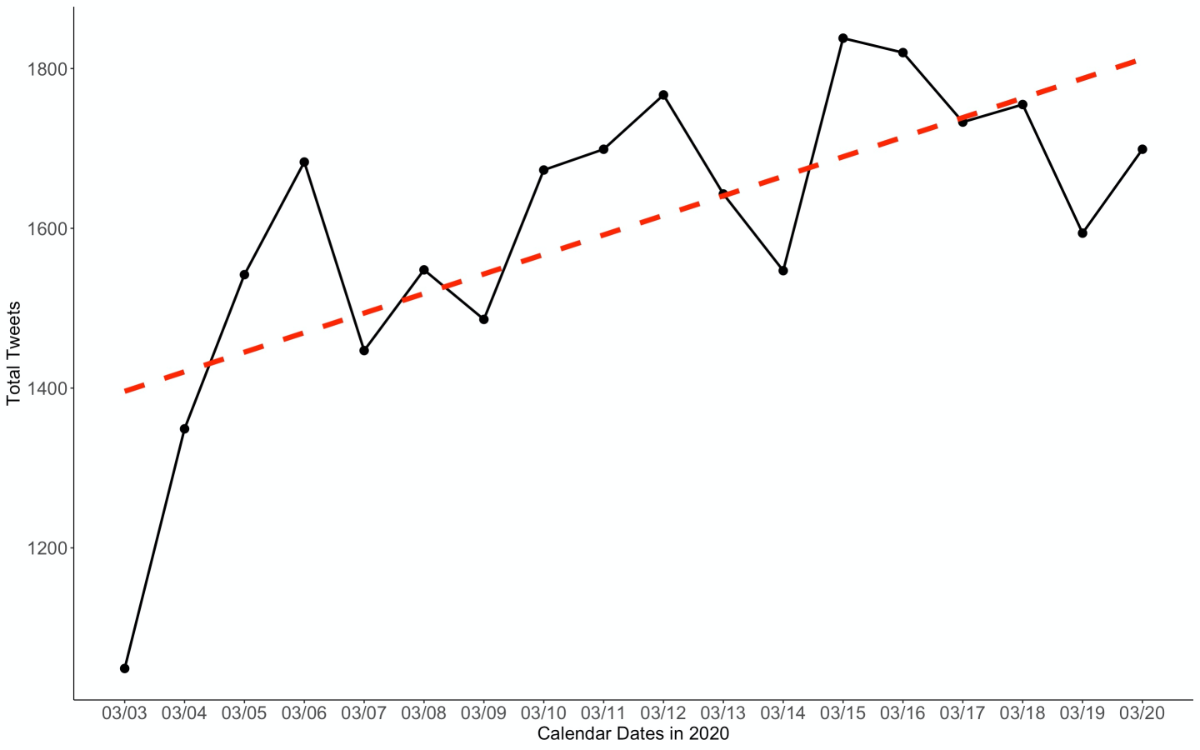
Volume of total signal Twitter posts filtered for the coronavirus disease symptom terms plotted over the study period (March 3-20, 2020).

**Figure 2:**
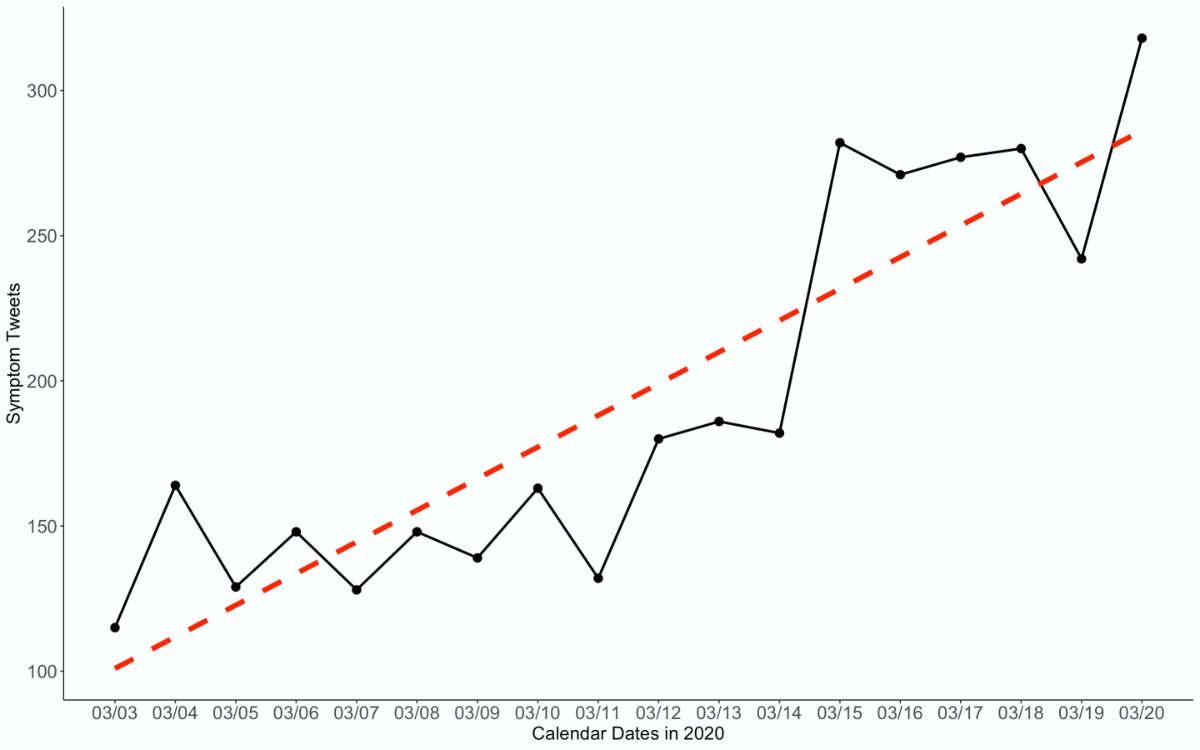
Volume of confirmed symptom tweets plotted over the study period (March 3-20, 2020).

In addition to content analysis, we assessed posts for descriptive longitudinal and geospatial trends by analyzing time stamps and location for the subset of tweets that were geotagged. Posts exhibited longitudinal trends with an overall increase during the study period, with noticeable rapid increases from March 3-6, 2020, and an uneven but gradual increase thereafter (Figures 1 and 2). Out of the 35,786 extracted tweets from the BTM topic clusters, 1769 (4.94%) included geospatial coordinates compared to 522,958 (0.71%) and 22,048 (0.49%) tweets that had coordinates in the entire corpus and the term-filtered data set, respectively. Hence, our total corpus is similar to other studies, reporting that approximately 1% of all tweets were geotagged, and our BTM topic cluster output had an overall higher volume of geolocated tweets in its sample [27,28]. From a global standpoint, 64.9% (n=1125) originated from the United States, followed by the United Kingdom (n=228, 13.2%), Canada (n=52, 3.0%), India (n=52, 3.0%), and Australia (n=43, 2.5%).

The high presence of US-based tweets and tweets from countries where the majority language is English (with the exception of India) is likely reflective of our sampling methodology, which focused on English-language tweets, and the fact that the highest proportion of Twitter users are located in the United States. This skewed geographic global distribution of tweets has also been explored in other studies that found a small number of countries (led by the United States) that account for a large share of the total Twitter user population [29]. The practical implications of this US-skewed geotagging mean that it is likely difficult to infer geospatial trends for tweets on specific COVID-19-related topics for other countries unless data collection is more targeted (eg, collection of tweets in foreign languages, in a specific time zone, or targeting geotagging for country or region-specific shapefiles).

**Figure 3:**
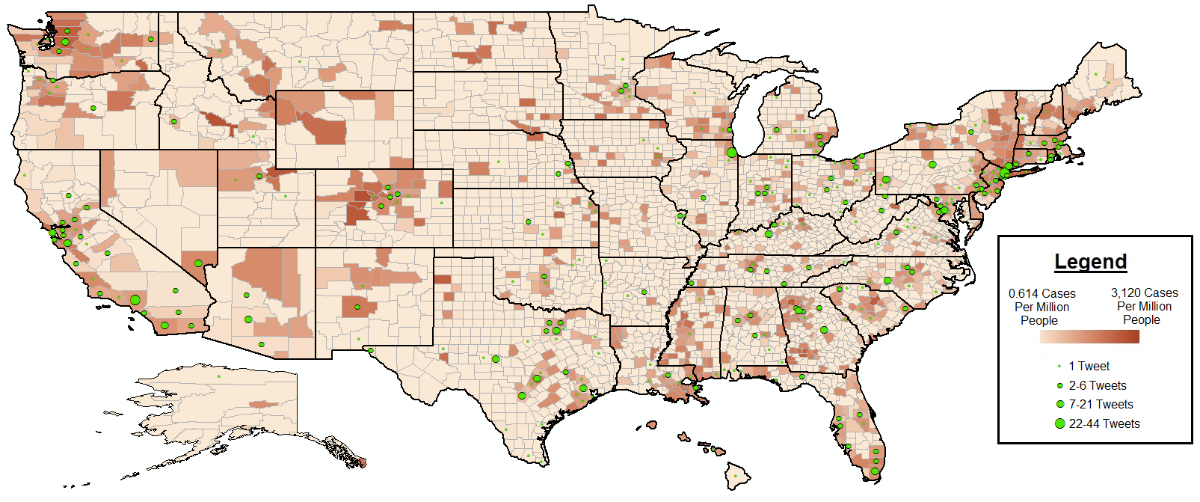
Distribution of tweets originating from the United States as point coordinates overlaid on a choropleth gradient denoting population-normalized coronavirus disease cases on March 20, 2020 (final day of data collection).

From a national perspective, the US states with the most tweets associated with COVID-19 symptoms and disease experiences were California (n=165), Texas (n=126), New York (n=88), and Illinois (n=54), which largely follows the most populous states in the country (with the exception of Florida). Manual coding revealed a similar ranking of symptom-related tweets that mentioned a state or city as self-reported by the user: California (n=43), New York (n=33), Texas (n=31), and Georgia (n=16). Even though these tweets had the highest frequency in larger states, smaller states and states that had reported confirmed COVID-19 cases (eg, Washington State) were also detected. Overall, COVID-19 associated symptom tweets exhibited wide distribution of Twitter user locations, including many in areas with high levels of population-normalized COVID-19 confirmed case counts (Figure 3).

Spearman correlations were also computed between the following variables associated with tweets collected: conversations about symptom reporting, experiences with lack of testing, recovery from suspected symptoms, and US location to assess co-occurrence of detected themes. Statistically significant positive correlations exceeding *r*=0.3 were observed between tweets that included users self-reporting symptoms and experiences with lack of testing (*r*=0.33, *P*<.001), as well as self-reporting of symptoms and self-reported recovery from reported symptoms (*r*=0.45, *P*<.001).

## Discussion

### Principal Findings

This study identified tweets that included both first- and secondhand self-reporting of symptoms, lack of access to testing, and discussion of recovery that users associated with possible COVID-19. The total volume of these COVID-19 conversations increased over the time of the study (particularly between March 3 and March 6, 2020), also corresponding with a period that saw an increase in the number of confirmed cases in the United States. The majority of these conversations related to first- or secondhand reporting of symptoms users associated with COVID-19, with a subset of this group concurrently reporting that they could not get access to testing despite having COVID-19-related symptoms. Other topics that occurred in lower frequency included self-reported recovery from symptoms, users confirming they were not COVID-19 positive, and past accounts of symptoms users believed could have been undetected cases of COVID-19 (dating back as early as November 2019).

Correlation analysis of themes generated by different tweets analyzed for this study found that it was more likely that users who self-reported symptoms they associated with COVID-19 would also concurrently report experiences with lack of access to testing or recovery from said symptoms. These results indicate that the public’s lived experience with COVID-19 included uncertainty about whether they or others were infected with COVID-19, frustration that they could not get tested to confirm these concerns, and sometimes their recovery experience from these symptoms. However, this study was not able to confirm if users reporting these experiences were actually COVID-19 cases, and users may similarly have not tweeted if they had eventually received confirmatory testing or otherwise if there was a change in their condition.

Importantly, ascertaining accurate case estimations of the COVID-19 outbreak is critical to ensuring health care system capacity is not overburdened; evaluating the impact of public health interventions; better enabling comprehensive contact tracing (including methods of digital contact tracing); ensuring the accuracy and predictability of COVID-19 disease mathematical modeling; and assessing the real-world needs for COVID-19 treatment, medical equipment, diagnostics, and other supplies [30,31]. Other online tools, such as the website COVID Near You [32], have collected self-reported symptom and testing access data directly from the public to better inform these case estimations.

Relatedly, the value of our study is in its innovative approach using data mining in combination with modeling to sift through a large volume of unstructured data to detect and characterize potential underreported cases of COVID-19. The methodology has particular utility for new and emerging topics such as a novel infectious disease outbreak where an existing training or labelled data set is not available for machine learning classification tasks. Specifically, our study tapped into an existing publicly available data source to help characterize conversations from Twitter users about their self-reported experiences with COVID-19 and provides insight into one period of this evolving and rapidly spreading global pandemic. It is our hope that this study can help inform future infoveillance efforts, supplement traditional disease surveillance approaches, and advance needed innovation to improve the scope and accuracy of future disease outbreak case estimations for COVID-19 and future health emergencies.

### Limitations

This study has limitations. We only collected data from one social media platform and limited study keywords and additional filtered terms to the English language. This likely biased study results to English speakers and primarily English-speaking countries, particularly since the highest number of Twitter users are already located in the United States. In fact, in our final data set of signal tweets, we did not observe any conversations in languages other than English. Our keywords and filtered terms were also chosen on the basis of our own manual searches on the platform but may not have been inclusive of all Twitter conversations related to the study aims. Future studies should expand data collection and analysis approaches to different languages and phrases associated with COVID-19 symptoms, testing, and recovery to obtain a more worldwide representative corpus of social media conversations. We also did not cross-validate the veracity of user-generated comments with other data sources (eg, confirmed case reports, additional survey data, death certificates, data on other diseases with similar symptoms, or electronic medical records). Future studies should explore combining multiple data layers from different sources to better validate whether user-generated self-reporting is highly associated with confirmed cases, case clusters, and disease transmission trends using traditional, syndromic, and other infoveillance approaches while also controlling for seasonal incidence of symptomatically similar diseases (upper respiratory infections, pneumonia, and flu or influenza). Additionally, though we used data filtering and BTM to more efficiently analyze a large corpus of tweets, we nevertheless relied on manual annotation to confirm whether tweets contained a signal. This was particularly important to remove false positives generated by our BTM outputs (ie, the word “testing” can take on different meaning depending on the context of a conversation). Future studies should also focus on developing feature-based supervised machine learning classifiers based on identified conversation characteristics reported in this study to detect self-reported COVID-19 experiences with symptoms, testing, and recovery. Specifically, supervised models that can leverage validated training sets are likely to have a much higher performance in terms of precision and recall compared to the use of topic models used in this study and could likely achieve classification closer to real time. Given that accurate case estimations are more effective when they are timely and can be acted upon quickly, these future approaches would likely have more utility in aiding with unreported case detection, identifying potentially vulnerable or at-risk populations, and better elucidating the public’s lived experiences with COVID-19.

## Appendix

Multimedia Appendix 1 Coding methodology for content analysis.

## Abbreviations

API: application programming interface
BTM: biterm topic model
COVID-19: coronavirus disease
ER: emergency room
