# Supplementary material for: Machine Learning to Detect Self-Reporting of Symptoms, Testing Access, and Recovery Associated With COVID-19 on Twitter: Retrospective Big Data Infoveillance Study
Source: JMIR Public Health Surveill. 2020 Jun 8;6(2):e19509. doi: 10.2196/19509 (PMC7282475; doi:10.2196/19509)
Supplement: Multimedia Appendix 1 [file publichealth_v6i2e19509_app1.docx]

**Supplemental file 1: Coding Scheme for Tweets**

Manual annotation of Tweets: The following coding scheme was used for binary classification of whether the tweet discussed COVID-19 symptoms (including first-hand or second-hand accounts), testing access experiences, or disease recovery, and co-occurrence of these themes.

| **THEME** | **SIGNAL** | **CODING SCHEME** |
| --- | --- | --- |
| **Symptom Reporting** (First-hand/ Second-hand) | YES | - Mention of specific flu-like symptoms (fever, cough, headache, other respiratory symptoms) - Other symptoms associated with COVID-19 (loss of taste/ loss of smell) - Reporting current symptoms - Reporting past symptoms (November 2019 – March 2020) - Reporting symptoms of self or friends/family/neighbors (mention of relationship, tagging the profile of other users) - Reporting visiting clinic/urgent care/ER for apparent COVID-19 symptoms - Reporting hospitalization due to pneumonia like conditions with flu test negative |
|  | NO | - Tweeting news posts - Pictures of symptoms - Discussing symptoms of COVID-19 to create awareness - Reporting symptoms prior to November 2019 - Sarcasm/jokes about COVID-19 symptoms - Reporting symptoms of celebrities/other public figures - Tweeting a list of COVID-19 symptoms from WHO, CDC etc., |
| **Lack of access to testing** | YES | - Reporting inability to get tested despite persisting symptoms (self-reporting and about friends/family/neighbors) - Tweeting about calling hospitals/clinics for testing appointments and being denied testing access - Discussing having symptoms and “no testing sites” in a respective community or neighborhood - Reporting not being tested for COVID-19 after negative flu test and persisting symptoms |
|  | NO | - Tweeting news alerts - Tweeting about the mention of “lack of testing” by politicians/health officials/government officials - Sarcasm/jokes on COVID-19 testing |
| **Reporting recovery from symptoms** | YES | - Tweets on recovering from symptoms that first appeared during the outbreak period (self-reporting and about friends/family/neighbors) - Reporting improvement post-hospitalization/ post-medication |
|  | NO | - Tweeting news alerts - Sarcasm/jokes |
